# Primary Step Towards In Situ Detection of Chemical Biomarkers in the UNIVERSE via Liquid-Based Analytical System: Development of an Automated Online Trapping/Liquid Chromatography System

**DOI:** 10.3390/molecules24071429

**Published:** 2019-04-11

**Authors:** Thomas Ribette, Bertrand Leroux, Balkis Eddhif, Audrey Allavena, Marc David, Robert Sternberg, Pauline Poinot, Claude Geffroy-Rodier

**Affiliations:** 1 Institut de Chimie des Milieux et Matériaux de Poitiers (IC2MP), Université de Poitiers, UMR CNRS 7285, Equipe Eau Géochimie Santé, 4 rue Michel Brunet, 86076 Poitiers, France; thomas.ribette@univ-poitiers.fr (T.R.); Bertrand.leroux@univ-poitiers.fr (B.L.); balkis.eddhif@univ-poitiers.fr (B.E.); audrey.allavena@univ-poitiers.fr (A.A.); pauline.poinot@univ-poitiers.fr (P.P.); 2 Laboratoire Interuniversitaire des Systèmes Atmosphériques (LISA), Université Paris Est Creteil, UMR CNRS 7583, 61 avenue du General de Gaulle, 94010 Créteil, France; marc.david@lisa.u-pec.fr (M.D.); robert.sternberg@lisa.u-pec.fr (R.S.)

**Keywords:** space instrumentation, liquid chromatography, oligopeptides, trapping system

## Abstract

The search for biomarkers in our solar system is a fundamental challenge for the space research community. It encompasses major difficulties linked to their very low concentration levels, their ambiguous origins (biotic or abiotic), as well as their diversity and complexity. Even if, in 40 years’ time, great improvements in sample pre-treatment, chromatographic separation and mass spectrometry detection have been achieved, there is still a need for new in situ scientific instrumentation. This work presents an original liquid chromatographic system with a trapping unit dedicated to the one-pot detection of a large set of non-volatile extra-terrestrial compounds. It is composed of two units, monitored by a single pump. The first unit is an online trapping unit able to trap polar, apolar, monomeric and polymeric organics. The second unit is an online analytical unit with a high-resolution Q-Orbitrap mass spectrometer. The designed single pump system was as efficient as a laboratory dual-trap LC system for the analysis of amino acids, nucleobases and oligopeptides. The overall setup significantly improves sensitivity, providing limits of detection ranging from ppb to ppt levels, thus meeting with in situ enquiries.

## 1. Introduction

The search for traces of past or present life in the solar system arouses the curiosity of many scientists. Detection of organic biomarkers has become a key challenge in planetary exploration, in order to understand whether they played a role in the origin of life on Earth [1,2]. Taking Earth-based techniques to develop a spatial instrument suite with multiple capabilities is, however, a real challenge. In space, even simple chemical analyses involve complex sample handling inside a probe’s instrument systems and require a sophisticated design with mass and power constraints as major factors. Within this framework, the technique of choice which was, and is still, employed for landed missions dedicated to the quest of life traces is pyrolysis gas chromatography mass spectrometry (Py-GC-MS) [3]. Based on a compound’s volatility, it was designed to detect low- to intermediate-molecular-weight organic biomarkers with improved sensitivity, leading to the in situ detection of organic molecules, as demonstrated by the Rosetta mission [4,5,6,7]. From Viking to the next ExoMars 2020 mission, this approach has been greatly improved with the use of online chemical derivatization agents, multi-column chromatographs and integrated traps (tenax, carbosieve, glass bead) [3,4,8,9,10,11,12]. These onboard instruments enable the detection of organic molecules, such as polycyclic aromatic hydrocarbons (PAHs) [13], amino acids [14,15] and sugars [16,17]. While such molecules have already been found in the interstellar medium and in many meteorites, their detection on extra-terrestrial planets remains a difficult task, and their assignment to a definite biological origin is still questioned, as they can be abiotically produced. To find additional data leading to unambiguous features of extant or extinct life and/or of prebiotic chemistry beyond Earth, researchers now pay attention to molecular biological polymers [18]. As a consequence, future onboard instruments must be able to detect and quantify all these compounds in one pot. As solid, liquid and aerosol samples are anticipated, the future instrument platform should involve versatile sample analysis. Gas and/or vapor samples can be analyzed directly by mass spectrometers or gas chromatograph-mass spectrometers [19,20], which have already been successfully used in previous planetary [11,21] and cometary missions [10,22,23]. Liquid (lake, icy regolith/cryovolcanic meltwater) and solid samples, undergoing melting, extraction or solubilization into a liquid mobile phase before analysis, could be analyzed either by gas chromatography (GC) or liquid chromatography (LC). In recent years, micro-fluidic systems involving sandwich and/or competitive immunoassays [24,25], microchip capillary electrophoreses [26,27,28] or nanopore-based analysis [29] have been designed [30]. A wide range of molecular-sized compounds, from amino acids and nucleobases to oligopeptides and oligonucleotides, would then likely be detected. Although these methods have already demonstrated real benefits in terms of sensitivity (1 µM to 0.1 nM) [31,32], some problems remain unresolved, even on Earth. Firstly, only a few molecules can be analyzed simultaneously, compared to multidimensional methods used in laboratories, e.g., two-dimensional gel electrophoresis (2D-PAGE) or two-dimensional liquid chromatography (2D-LC) [28]. Another major hurdle is the very small sample volume that can be analyzed in one run (from 1 nL to 10 µL [31]), which reduces the method’s sensitivity and sample representativity. In addition, only a pure extract can be injected in these micro-fluidic systems; as a result, they require a previous complex and multi-step off-line sample preparation [25,27,33,34,35]. Thanks to remarkable improvements in multidimensional systems and UPLC stationary phases, an online analytical platform allowing both the purification and analysis of a various set of compounds could now be considered for exobiological studies. 

The aim of the present study is to develop a liquid setup able to concentrate and separate a wide range of potential extra-terrestrial peptide-like molecules. The developed configuration, in addition to its intrinsic qualities, such as the concentration and great versatility despite the drastic operating conditions, would have to facilitate a simple and fast separation suitable for in situ analysis. Placed online with a spatialized detector [36,37], the generic unit should potentiate the one-pot detection of diverse molecules with increasing complexity and present at nanomolar or picomolar levels. 

MS detectors, coupled to liquid chromatography, have already allowed major advances in organics characterization of meteorite, tholin and comet analogues [38,39,40]. Thanks to a Q-Orbitrap High Resolution Mass Spectrometer (HRMS) that allows a comprehensive assessment of the data obtained, several analytical features were assessed to define the best trapping and separation conditions. Finally, the approach, combined with a simplified sample preparation protocol and spatialized detector, could potentially be validated for space life-search experiments. 

## 2. Materials and Methods 

### 2.1. Chemicals and Solutions

Several monomers and polymers considered to be strong biosignatures of life (amino acids, nucleic acids and oligopeptides) were used at different stages of optimization, as they are distinct in terms of polarities, chemical structures and molecular masses, and such biomarkers were used to select generic trapping parameters. 

A commercial mix of five oligopeptides (Sigma-Aldrich, Steinheim, Germany) contained a dipeptide (glycine-tyrosine (gly-tyr)), a tripeptide (valine-tyrosine-valine (val-tyr-val)) and three oligopeptides (leu-enkephalin, met-enkephalin and angiotensin II), each at 0.5 mg. Nucleic acids such as cytosine (>99%), uracil (>99%) and thymine (>99%) were purchased from Sigma-Aldrich. Amino acids (alanine, glycine, valine, leucine, isoleucine, proline, methionine, arginine, cysteine, threonine, serine, aspartic acid, glutamic acid, histidine, lysine, phenylalanine, tyrosine) and other oligopeptides were supplied by Sigma-Aldrich (St. Louis, MO, USA). LC mobile phases were prepared with HPLC grade acetonitrile and ultrapure grade formic acid, purchased from Sigma-Aldrich. Purified water was generated by a Purelab Flex purifier system (Veolia, Paris, France).

### 2.2. Preparation of Standard Solutions

Two stock solutions were prepared with oligopeptides solubilized in high purity water, amino acids in HCl 1 M and nucleobases in NaOH 0.1 M The first solution contained the peptides (0.5 µg/mL) in high-purity water, the second was composed of the amino acids and nucleobases (1.5 × 10^−6^ M). The standard solution containing the oligopeptides was used to prepare a working solution at 0.01 µg·mL^−1^, which was used to select the optimal trapping parameters of the trapping-LC setup in comparison with the 1D-LC method. This solution was further mixed and diluted with the standard solutions, containing amino acids and bases, to prepare the different series of calibration solutions to validate the optimized system. 

### 2.3. 1D-LC Setup

The analysis was performed with a Wadose LC isocratic pump, interfaced with a Q-Exactive Hybrid Quadrupole-Orbitrap mass spectrometer equipped with an ESI source (Thermo Fisher Scientific, Waltham, MA, USA). The MS functions were controlled by the Xcalibur data system (Thermo Fisher Scientific), whereas injection and HPLC solvent elution were monitored and controlled by our software developed on LabVIEW. The analytical column was a semi-polar Hypersil Gold aQ (50 × 1 mm, 1.9 µm, 175 Å, Thermo Fisher Scientific). The mobile phase consisted of acetonitrile (ACN)–0.1% formic acid and water–0.1% formic acid. Elution was performed with 10% and 20% ACN at a constant flow rate of 110 µL·min^−1^. Experiments were conducted at 40 °C.

### 2.4. Trapping-LC Setup

The analysis was performed with the same instrumentation. The pump enabled the loading of the sample on the trapping setup, followed by the backflush and the analytical separation of analytes. Two different trapping columns, a semi-polar Hypersil Gold aQ (20 × 2.1 mm, 12 µm, 175 Å; Thermo Fisher Scientific) and a polar Hypercarb (20 × 2.1 mm, 7 µm, 175 Å; Thermo Fisher Scientific), were used. 

One thousand microliters of the sample was injected in the preparative loop. The compounds were transferred to the trapping columns at 500 µL·min^−1^ for 180 s. Non-retained compounds (e.g., matrix interferences, salt) were flushed to waste. Once the loading completed, trapped analytes were backflushed at 110 µL·min^−1^ until all targets were eluted on the analytical column. During this time, trapping and analytical columns were connected in series and eluted by means of the 1D-LC mobile phase. The valve scheme is described in Section 3.3. The automation was performed by LabVIEW® software (version 2016, National Instrument Corporation, Austin, TX, USA) that controlled the pump, valves and column oven. 

The system was compared to a laboratory dual-trap system with two quaternary Accela LC pumps (600 and 1250) working together and interfaced with the same Q-Exactive Hybrid Quadrupole-Orbitrap Mass Spectrometer. The Accela 600 pump provided the loading of the sample on a trapping-LC unit, while the 1250 pump controlled the backflush and the analytical separation of analytes. Before injection, samples were stored at 4 °C using a Stack cooler CW (CTC Analytics AG, Zwingen, Switzerland). MS functions and HPLC solvent gradients were controlled by the Xcalibur data system (Thermo Fisher Scientific).

### 2.5. Mass Spectrometry

The analysis was carried out on a Q-Exactive mass spectrometer. Mass detection was performed in positive ion mode. The electrospray voltage was set at 4.0 Kv. The capillary and heater temperatures were 275 °C and 300 °C, respectively. The sheath, sweep and auxiliary gas (nitrogen) flow rates were set at 35, 10 and 20, respectively (arbitrary units).

MS analyses were performed by either full scan or targeted selected ion monitoring mode (tSIM). The full scan mode was employed when standard solutions were analyzed. Mass spectra were acquired at 70,000 resolution, AGC target 10^6^ and max IT 200 ms. Compounds were analyzed in the range of 300–2000 *m*/*z* when solutions of oligopeptides were analyzed (i.e., solutions used for the selection of optimal multidimensional parameters), and in the range of 75–1100 *m*/*z* when solutions contained amino acids and nucleobases (i.e., calibration solutions).

tSIM MS offered superior sensitivity when complex samples were analyzed. It was then used to determine the recovery of compounds with resolution at 17500, AGC target at 10^5^, max IT at 200 ms and MSX count at 4. Detection of organics was set within 2 ppm of the theoretical mass. 

Leu-Leu *m*/*z* 245.18657 [M + H]^+^; Leu-Leu-Leu *m*/*z* 358.27003 [M + H]^+^, Phe-Phe *m*/*z* 312.36 [M + H]^+^; Met-Met-Met *m*/*z* 412.13985 [M + H]^+^; Gly-Tyr, *m*/*z* 239.10263 [M + H]^+^; Val-Tyr-Val, *m*/*z* 380.21747 [M + H]^+^; Gly-Gly-Gly *m*/*z* 190,08281 [M + H]^+^; Ala-Ala-Ala-Ala, *m*/*z* 303.16685 [M + H]^+^ Leu-enkephalin, *m*/*z* 556.27658 [M + H]^+^; Met-enkephalin, *m*/*z* 574.23300 [M + H]^+^; Angiotensin II, *m*/*z* 523.77453 [M + 2H]^2+^; Guanine, *m*/*z* 152.0567 [M + H]^+^; Thymine, *m*/*z* 127.05669 [M + H]^+^; Cytosine, *m*/*z* 112.05054 [M + H]^+^; Uracil, *m*/*z* 113.03512 [M + H]^+^.

## 3. Results and Discussion

### 3.1. UPLC General Features 

In this rationale, peptides were used as molecular targets. To overcome the lack of extra-terrestrial peptide standards, amino-acids polymers differing in terms of molecular weight and polarity were considered [33]. Oligopeptides with alanine, glycine and leucine were considered as valuable targets, since their building blocks are the main amino acids in the acid hydrolysates of meteorites, tholins and comets [22,41,42]. The sensitivity of the system was thus investigated based on concentrations of meteorite compounds. Amino acid and nucleobase concentrations present in carbonaceous meteorites range from ppb to ppm levels (ng·g^−1^ to µg·g^−1^ of meteorite) [15,43]. Assuming a similar range in the universe, the limit of detection of any technique used in situ has to be at least at the ppb level. For a 1 g sample of liquid, melted or extracted in 1 mL of solvent, a detection limit of ng·mL^−1^ is mandatory. 

To develop a simple but efficient liquid chromatographic system for space experimentation, a single isocratic pump was used to perform the trapping and separation of both compounds. For that purpose, a minimum length of flexible stainless steel capillaries together with Viper Fingertight Fitting system were selected to provide virtually dead-volume-free plumbing, minimizing extra-column dispersion. 

Stationary phases were chosen according to their relevance for peptide-like compound analysis. Alone and in series, short columns (20–150 mm) with stationary phases exhibiting different polarities were previously evaluated in gradient mode for the analysis of laboratory cometary analogues [33]. Briefly, in isocratic mode, elution on Reverse Phase C_18_ Hypersil Gold aQ, with acetonitrile as the organic solvent, allowed the study of complex mixtures with high peptide retention. To decrease the mobile phase consumption and increase the sensitivity, a low-diameter (1 mm) Hypersil Gold aQ column was selected as the analytical column. To ensure the high solubility of the oligopeptides in the mobile phase, with the lower energy consumption of the column oven, the temperature was set at 40 °C.

Automation and control (oven, pump and valves) were performed by an interface programmed on LabVIEW.

### 3.2. 1D-LC Configuration

**Figure 1:**
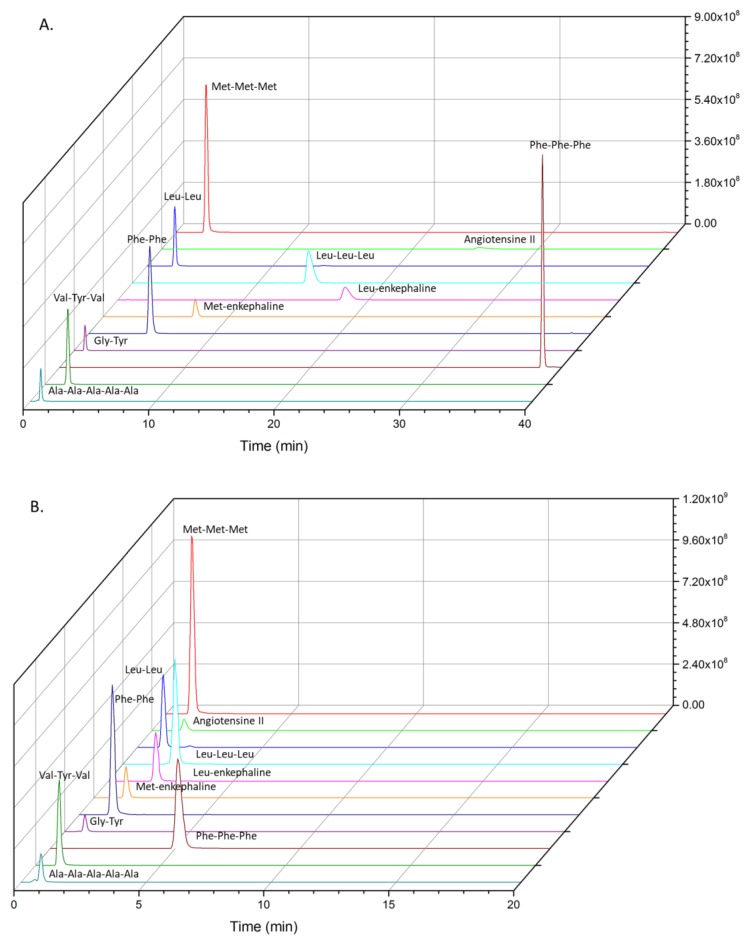
Direct injection extracted ion chromatogram: 20 µL of peptides solution (0.5 µg/mL) eluting on Hypersil Gold aQ (50 mm × 1 mm, 40 °C) with (**A**) 10/90 acetonitrile (ACN)/H_2_O and (**B**) 20/80 ACN/H_2_O as the mobile phase.

For direct injection, best separation was achieved with a 10/90 ACN/H_2_O mobile phase, regarding intensities and retention times of the different oligopeptides (Figure 1, Table 1). 

Increasing the volume of injection would be a way to improve sensitivity as a slight amount of complex and highly diluted sample is expected to be available [44]. An online liquid-trapping system would then be necessary to enable a large volume injection, to clean up samples (highly aqueous, salts-containing, etc.) and to selectively trap molecules of interest.

### 3.3. Trapping-LC Configuration

Regarding space constraints, trapping must be performed under an unusual configuration with a single pump for trapping and elution. 

Various trapping factors, such as column stationary phases, loading and backflush parameters, strongly influence compound recovery and cleanup efficiency [45,46,47]. Stationary phases of the trapping columns were previously selected to characterize high-molecular-weight compounds in a cometary ice analogue. Briefly, Hypersil Gold aQ allowed the retention of semi-polar and apolar peptides, while more polar and low-mass compounds were refocused at the head of a Hypercarb column. By serially coupling both columns and setting a loading flow rate of 500 µL·min^−1^ for 120 s and a backflush of 240 s, this dual-trap setup led to the best retention of all standards [33].

**Figure 2:**
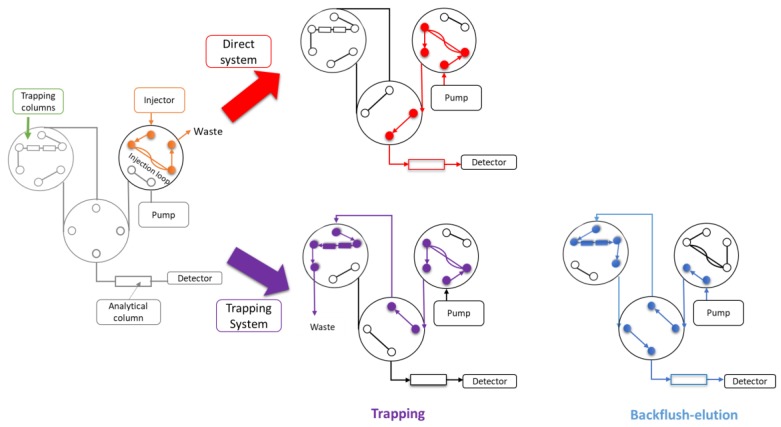
Schematic representation of the direct and trapping-LC setups.

To adapt this system to in situ analysis, the laboratory loading pump was suppressed and a switching valve was added (Figure 2). The designed system was thus composed of two trapping columns coupled to the analytical dimension. In that configuration of a single pump, the backflush step corresponded to elution on the analytical column. The only parameter to be optimized was then the nature of the mobile phase. To evaluate the system, trimethionine was chosen as an internal standard.

**Figure 3:**
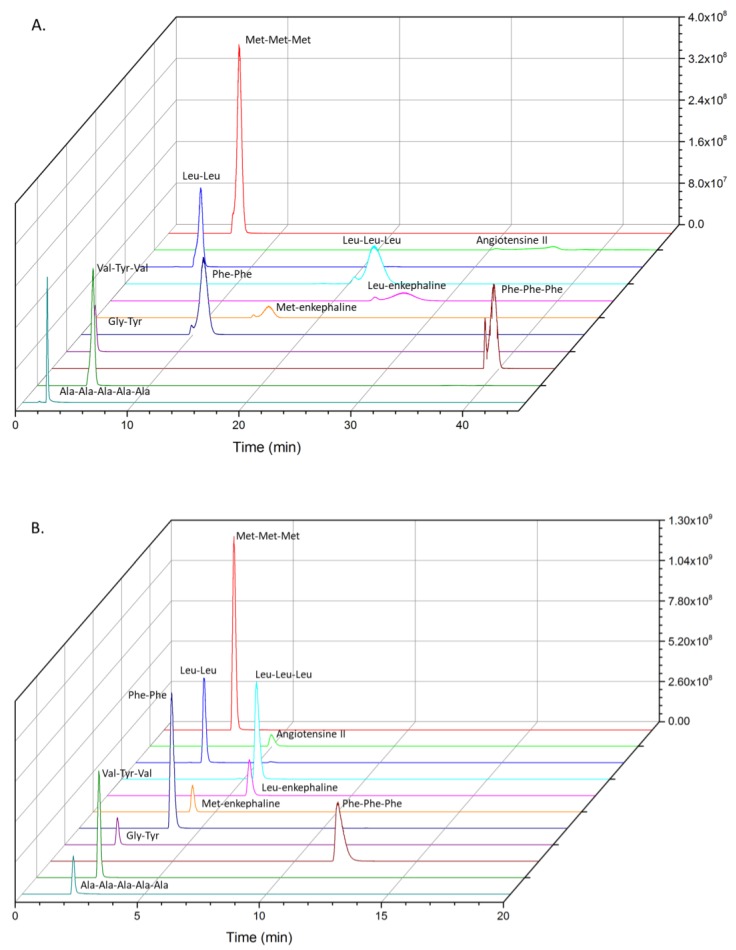
Extracted ion chromatogram of trapped peptides (0.5 µg/mL) on Hypersil Gold aQ (50 mm × 1 mm, 40 °C). Backflush-elution with (**A**) 10 % ACN and (**B**) 20% ACN.

**Figure 4:**
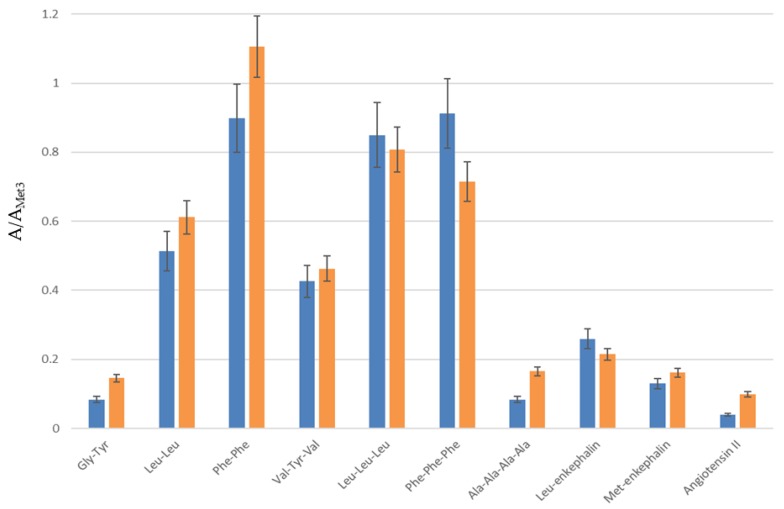
Recoveries of trapped peptides from a standard sample 1.5 × 10^−6^ M (0.5 µg/mL) after backflush and isocratic elution with 10% (in blue) or 20% ACN (in orange) on Hypersil Gold. Results are given as the ratio of compound area over internal standard one (A/A_Met3_).

Peaks tailing and broadening of the highest molecular weight oligopeptides with 10% ACN were not suitable for the elution of non-targeted oligopeptides. Backflush and elution with 20% ACN gave, on the contrary, a real benefit in terms of separation, as less coelution occurred for the studied peptides compared to the direct injection 1D-LC configuration (Figure 1 and Figure 3). On the whole, peaks were well-defined. The delay of 180s in elution was particularly interesting for polar and/or very-low-molecular-weight compounds, which were no longer eluted at the death retention time. Backflush with 20% ACN gave also the best recoveries, except for phenylalanine tripeptide (loss of 22%, Figure 4).

### 3.4. Interest of the Trapping-LC Setup for In Situ Experiments

Under space constraints, time and solvent consumption have to be considered. In our configuration, if LC was chosen to be part of the on-board instrumentation, it would analyze samples in less than 20 min with 2 mL of a single mobile phase. These features comply with in situ conditions and constitute a good basis for future improvements.

**Figure 5:**
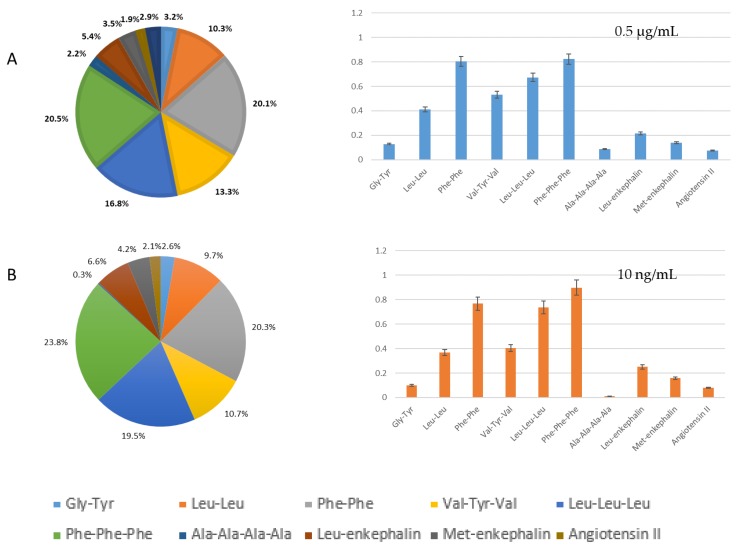
Repartition and recoveries of each peptide (**A**) in direct injection configuration 0.5 µg/mL and (**B**) in trapping configuration 10 ng/mL. Results are given as the ratio of compound area over internal standard one (A/A_Met3_).

The retention capability of our designed system was compared to direct injection without trapping. The performance of the system was evaluated by injecting the same number of oligopeptides in direct (20 µL, 0.5 µg/mL) and trapping configurations (1000 µL, 10 ng/mL). As illustrated by Figure 5, there was no major difference in peptide retention and detection. Both distributions of oligopeptides were similar. The trapping was, however, not efficient for all the peptides, as alanine one was not retained. 

The trapping-LC system led, however, to significantly higher signal intensities as similar responses were obtained with a 50-fold lower concentration in trapping configuration. 

**Figure 6:**
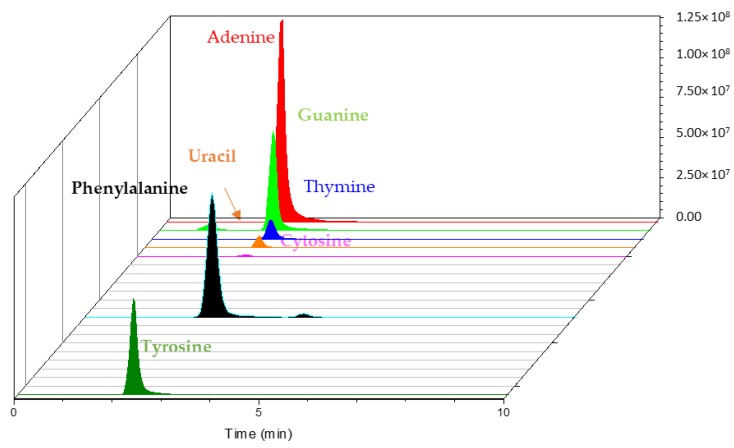
Extracted ion chromatogram of trapped amino acids and nucleobases on Hypersil Gold aQ (50 mm × 1 mm, 40 °C) from a 1 mL water sample. Backflush-elution with 20% ACN.

Regarding targets for future space exploration missions, amino acid and nucleobase trapping was then evaluated. Contrary to nucleobases, in the optimized peptide trapping conditions, no amino acid was retained, except for phenylalanine and tyrosine (Figure 6). 

**Figure 7:**
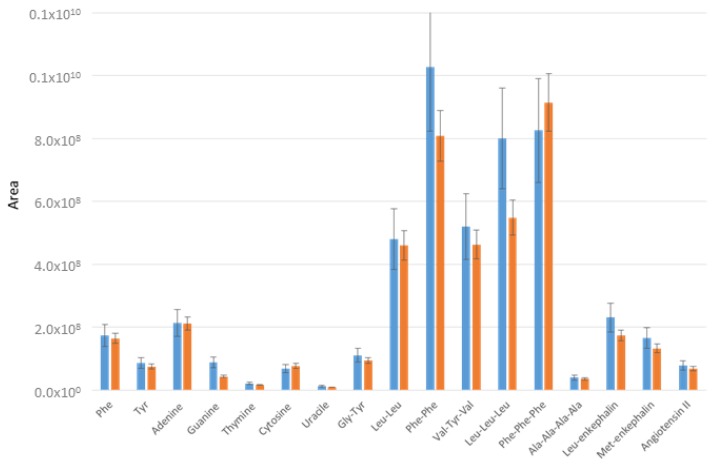
Recoveries of trapped molecules from a standard sample 1.5 × 10^−6^ M (0.5 µg/mL), with the trapping-LC system in blue and a laboratory dual-trap LC system after backflush and isocratic elution with 20% ACN on the Hypersil Gold column.

Analyses were then performed with a laboratory dual-trap system. Figure 7 shows the responses of the two trapping systems for retained amino acids, bases and oligopeptides. For all the targets, similar retention was obtained but with a higher standard deviation for the single-pump system (up to 26% for Phe-Phe). 

To further exemplify the sensitivity of the system when coupled to a mass spectrometer, the recovery of some targeted compounds was calculated using calibration curves (Table 2). Linearity ranged from 0.25 to 10 ng·mL^−1^.

Retained peptides, nucleobases and amino acids were detected at the ng·mL^−1^ level. This clearly demonstrates the effectiveness of this online trapping approach when highly diluted and complex samples are analyzed. This trapping unit, coupled to a liquid chromatography system, would then enlarge the set of data about potential exobiological molecules without denaturing them. Despite its ability to retain different organics in terms of polarity, chemical structure and molecular weight in a single run, this broad approach should also be able to raise the signal of highly diluted compounds. This is fundamental for liquid in situ experiments, since compound extraction would previously have to be reduced to an extreme simplicity with large volumes of final extracts (in the order of milliliters), and thus with a low recovery achievement. 

## 4. Conclusions

In situ detection of biomarkers in the solar system has become an appealing project, partly guiding past, present and future space missions. Up to now, in situ instrumentation was mainly designed to detect and determine concentrations of volatile organic compounds or derivatives. In this work, we present the first trapping unit for extra-terrestrial peptide-like compounds. The screening of several parameters showed that a trapping unit placed in series with an analytical column significantly enhanced the range of potential compounds to be analyzed. Through this LC setup, we do not pretend to separate all individual compounds in the sample as MD-LC systems do. Nevertheless, by avoiding mobile phase changes and reducing the system’s dead volume due to long tubing and viper connections, the chromatographic dilution of a compound’s band, as well as the tailing and broadening of peaks, are limited. As a result, the detection of very low concentrations of analytes is facilitated. Under space conditions, this system could present several advantages, since it would (1) elude chemical derivatization of non-volatile and polar compounds, as is necessary for current on-board GC-MS instruments, (2) limit the misinterpretation of chromatograms, (3) enlarge the range of potential biomarkers targeted and (4) reduce the complexity of offline sample preparation protocol used with microfluidic systems, without decreasing a compound’s signal intensity. It could then represent a powerful tool for exobiological studies.

## Figures and Tables

**Table 1:** Retention times (min) of oligopeptides eluted on Hypersil Gold aQ (50 mm × 1 mm, 40 °C) with 10/90 ACN/H_2_O and 20/80 ACN/H_2_O as the mobile phase.

Compounds	10% ACN	20% ACN
Ala-Ala-Ala-Ala	0.80	0.79
Gly-Tyr	0.85	0.80
Val-Tyr-Val	1.80	0.93
Leu-Leu	2.22	1.02
Met-Met-Met	2.39	1.01
Met-enkephalin	7.39	1.30
Phe-Phe	4,87	1.33
Leu-Leu-Leu	14.07	2.07
Leu-enkephalin	18.13	1.90
Angiotensin II	25.33	1.29
Phe-Phe-Phe	38.44	5.12

**Table 2:** Performance hallmarks of the designed trapping-LC system.

Compounds	R^2^	RSD% (*n* = 18 ^a^)	LOD ^b^ ng·mL^−1^	LOQ ^c^ ng·mL^−1^
Gly-Tyr	0.912	11.1	0.16	0.54
Val-Tyr-Val	0.983	10.3	0.11	0.36
Leu-Enkephalin	0.987	6.3	0.18	0.60
Angiotensin II	0.986	30.2	2.77	9.24
Phenylalanine	0.978	9.0	4.03	13.42
Tryptophan	0.976	16.6	3.08	10.27
Uracil	0.956	7.2	1.95	6.15
Cytosine	0.863 *	15.0	2.22	7.41
Thymine	0.965	10.3	1.85	6.18

^a^ Average relative standard deviation (six calibration points; three replicates per calibrant; *n* = 18). ^b^ Detection limit: 3.3 × (residual standard deviation/slope). ^c^ Quantification limit: 10 × residual standard deviation/slope). * Coefficient of determination and subsequent values (Relative Standard Deviation RSD, Limit of Detection LOD, Limit of Quantification LOQ) were below 0.9. Regression models were not validated.
